# Barriers to Investigator-Initiated Clinical Trial Enrollment in Frontline Large B-Cell Lymphoma

**DOI:** 10.1016/j.clml.2025.11.002

**Published:** 2025-11-04

**Authors:** Carrie Ho, Neena Kennedy, Mengyang Di, Ajay K. Gopal, Ryan C. Lynch, Kevin Ng, Qian V. Wu, Christina Poh, Vikram Raghunathan, Heather Rasmussen, Mazyar Shadman, Brian G. Till, Chaitra S. Ujjani, Stephen D. Smith

**Affiliations:** 1 Department of Internal Medicine, Division of Hematology and Oncology, University of California San Francisco, San Francisco, CA; 2 Division of Hematology and Oncology, University of Washington, Seattle, WA; 3 Fred Hutchinson Cancer Center, Seattle, WA

**Keywords:** Access, Clinical trials, Geography, Health disparities, Socioeconomic status

## Abstract

In this retrospective study of newly diagnosed LBCL patients referred to a tertiary cancer center, greater geographic distance and neighborhood socioeconomic deprivation were associated with lower enrollment in frontline investigator-initiated trials (IITs). The most common reason for trial ineligibility was initiation of treatment before referral, which disproportionately affected patients living farther from the center or in more socioeconomically deprived areas. These findings highlight persistent barriers to trial participation and support the need for earlier referral, telemedicine-based triage, and decentralized trial models to improve equity in access.

**Background:**

Frontline therapy fails to cure ~ 25% of patients with large B-cell lymphoma (LBCL), underscoring the need for clinical trials to improve outcomes. However, enrollment remains limited by logistical and structural barriers. Investigator-initiated trials (IITs) at our center enroll patients 5 to 10 times faster than industry-sponsored trials, yet the influence of geography and socioeconomic status on participation remains poorly understood.

**Methods:**

We retrospectively reviewed adults with newly diagnosed large B-cell lymphoma (LBCL) referred to Fred Hutchinson Cancer Center (FHCC) between October 2022 and June 2024. Patients were prescreened by a clinical research nurse and investigators for frontline IIT eligibility. Demographic, geographic, and socioeconomic data were collected, including sex, race, ethnicity, distance from FHCC, area deprivation index, insurance, and interpreter need. Logistic and elastic net regression were used to evaluate predictors of trial enrollment. Trial-ineligible patients were analyzed descriptively.

**Results:**

Of 153 patients, 68 (44%) were trial-eligible; 24 (35%) enrolled. Enrolled patients lived closer to FHCC (median 15 vs. 50 miles; *P* = .00015) and had lower ADI scores (median 8 vs. 21; *P* = .006) than non-enrolled eligible patients. In univariate analysis, both distance and ADI were associated with enrollment; in multivariable stepwise regression, only distance remained significant. Elastic net regression identified both distance and ADI as frequently selected predictors. Among 85 trial-ineligible patients, 61% had already initiated treatment; these patients also lived farther away and in more deprived areas.

**Conclusion:**

Geographic distance and neighborhood deprivation significantly influenced trial enrollment, even in investigator-initiated trials (IITs). Decentralized trial models, telemedicine triage, and system-level interventions are needed to reduce these inequities.

## Background

Despite advances in frontline therapy, approximately 25% of patients with newly diagnosed large B-cell lymphoma (LBCL) are not cured by first-line treatment. Clinical trials play a critical role in advancing cancer treatment and improving patient outcomes, yet equitable access to trials remains challenging. Additionally, only approximately 8% of U.S. cancer patients participate in clinical trials.^1^ Given that an estimated 70% of American adults express willingness or inclination to participate in a clinical trial, the stark discrepancy between willingness and actual trial enrollment rates suggests that systemic barriers continue to limit trial participation.^2^ Identifying and addressing these barriers is critical to improving trial accessibility and ensuring that novel therapies reach all patient populations, particularly those already experiencing disparities in outcomes.

Studies have shown that patients from low socioeconomic status (SES) areas experience poorer survival after LBCL, with disparities further exacerbated by race and insurance status.^3,4^ Black patients and those with Medicaid or no insurance face significantly worse outcomes.^5,6^ A large National Cancer Database study found that rural and urban patients with DLBCL and follicular lymphoma (FL) had worse survival compared to metropolitan patients, likely due to lower SES, reduced access to specialized care, and treatment at lower-volume centers.^4^

Given these disparities, ensuring equitable access to clinical trials is critical, as these studies provide early access to novel therapies and improve treatment options for high-risk populations. However, underrepresented populations, including racial minorities and socioeconomically disadvantaged patients, often face significant barriers to trial enrollment, potentially reinforcing existing disparities in LBCL outcomes. Trial enrollment remains disproportionately low among racial and ethnic minorities, lower-income individuals, rural patients, women, and the elderly.^7,8^ These findings suggest that socioeconomic deprivation may influence trial participation indirectly, through geographic barriers, financial constraints, and provider referral decisions.

At Fred Hutchinson Cancer Center (FHCC), trial accrual patterns provide a unique perspective on these barriers. Industry-sponsored trials typically enroll only 2 to 3 patients per site per year, likely reflecting challenges in patient eligibility and accessibility. In contrast, investigator-initiated trials (IITs) at FHCC accrue 5 to 10 times faster than industry-sponsored studies, likely due to their broader eligibility criteria, streamlined logistics, and greater local engagement. However, despite this higher enrollment rate, it remains unclear whether socioeconomic factors, geographic distance, and structural barriers continue to influence trial participation, even in this relatively optimized trial setting.

This study aims to identify the key barriers to trial enrollment in first-line LBCL IITs at FHCC. We hypothesize that geographic distance, socioeconomic deprivation, and patient demographics influence trial accrual, even within a setting with relatively high IIT enrollment. By systematically evaluating these barriers, we aim to inform strategies to improve access, optimize trial pathways, and promote equitable participation in LBCL clinical trials.

## Patients and Methods

We identified newly diagnosed adult patients with LBCL referred to FHCC in Seattle, Washington. These patients were pre-screened for clinical trials by a clinical research nurse and study investigators between October 2022 and June 2024. This start date was selected to coincide with the formal implementation of a dedicated clinical research nurse role, established to standardize and enhance the pre-screening process for clinical trials. Prior to this, pre-screening was performed ad hoc by study investigators and clinical research coordinators. During the study period, a clinical research nurse and study investigators systematically pre-screened all referred patients for two open IITs enrolling patients with newly diagnosed diffuse LBCL or high-grade B-cell lymphoma.

Demographic, geographic, and socioeconomic data were abstracted from the medical records, including sex, race, ethnicity, insurance type, interpreter need, and distance from the patient’s 9-digit residential ZIP code to FHCC. Socioeconomic disadvantage was measured using the 2022 area deprivation index (ADI), obtained from the Neighborhood Atlas.^9^ The ADI, a validated measure of neighborhood-level socioeconomic disadvantage at the census block group level, is based on factors such as income, education, and housing quality, and has been linked to disparities in cancer outcomes.^10–12^ Neighborhoods are scored on a percentile scale of 1 to 100, with lower scores indicating higher SES and higher scores reflecting greater deprivation.

We compared characteristics of trial-eligible enrolled and non-enrolled patients using the Wilcoxon rank-sum test for continuous variables and the chi-squared test for categorical variables. To assess associations between trial enrollment and factors including sex, age at screening, insurance status, distance from FHCC, ADI, race, and interpreter need, we conducted univariate logistic regression. Statistically significant variables (*P* < .1) were then incorporated into a stepwise multivariable analysis. To further evaluate the relative importance of predictor variables, we applied elastic net regression, a regularized modeling technique suited for smaller datasets and capable of handling correlations between predictors. The model was fit with a mixing parameter *α* = 0.9 and a minimum deviance λ selection criterion, using 100 iterations and 5-fold cross-validation. All predictors from the univariate models were included, and the frequency of nonzero coefficients was tabulated to assess variable stability. R software version 4.4.2 was used for statistical analysis.

We also assessed the diagnosis-to-treatment interval (DTI), defined as the number of days from diagnosis to initiation of first-line therapy. DTI was compared using pairwise Wilcoxon rank-sum tests: (1) between trial-eligible and trial-ineligible patients, and (2) enrolled versus not-enrolled patients within the trial-eligible group.

To visualize geographic patterns across key stages of the clinical trial accrual funnel, we generated county-level choropleth maps in R showing distributions of trial-ineligible, trial-eligible, enrolled, and not-enrolled patients. Counties with fewer than 20,000 residents were masked to protect patient confidentiality.

Finally, reasons for trial ineligibility were abstracted from clinical documentation, categorized, and summarized descriptively. This study was determined to be exempt from Institutional Review Board review and was conducted in accordance with the ethical principles of the Declaration of Helsinki.

## Results

### Patient Characteristics

A total of 153 patients with newly diagnosed LBCL were referred to our center and screened for clinical trial eligibility (Table 1). The cohort was predominantly male (61%), White (87%), and non-Hispanic or Latino (97%). Insurance type was primarily split between private (42%), Medicare (50%), and Medicaid (7%). Sixty-eight patients (44%) were deemed trial-eligible after initial pre-screening, and 24 (35%) of those enrolled, representing 15% of the total patients screened (Figure 1).

Characteristics of the trial-eligible enrolled vs not-enrolled groups are shown in Table 2. Trial-enrolled patients lived significantly closer to FHCC (median 15 miles, interquartile range [IQR] 7–18) than not-enrolled eligible patients (50 miles, IQR 19–109, *P* = .00015). Enrolled patients also had a lower ADI (median 8, IQR 4–14) compared to not-enrolled eligible patients (median 21, IQR 8–28, *P* = .006). All enrolled patients were non-Hispanic compared to 98% of non-enrolled patients, though this difference was not statistically significant. Age, sex, race, ethnicity, need for an interpreter, and insurance type were not significantly different between the enrolled and non-enrolled eligible groups.

### Univariate and Multivariate Regression Analysis

In univariate logistic regression, shorter distance to FHCC (*P* = .0269) and lower ADI (*P* = .0212) were significantly associated with trial enrollment. No other variables met the threshold for statistical significance (*P* < .1). In multivariable stepwise logistic regression, only distance remained a significant independent predictor (*P* = .0269) (Table 3). In contrast, elastic net regression identified both distance and ADI as the most frequently selected predictors of enrollment, appearing in 88% and 90% of models, respectively. No other variables were selected in more than 70% of models.

### Reasons for Trial Non-Enrollment and for Trial Ineligibility

Among 44 trial-eligible patients who did not enroll, the most common reason was patient preference due to geographic barriers (27%), followed by urgent treatment needs (10%), trial enrollment hold (5%), and desire to initiate earlier treatment (3%). The remaining 55% of patients received standard-of-care therapy based on unspecified patient or provider preferences; of these, 67% received treatment at FHCC and 33% at community practices.

Among 85 trial-ineligible patients, the median age was 68 years (range, 34–90), with 60% male, 88% White, and 96% non-Hispanic. The median distance from FHCC was 79 miles (IQR, 24–182), and the median ADI was 23 (IQR, 14–39). Insurance coverage included Medicare (53%), private insurance (39%), Medicaid (7%), and other government insurance (1%). The most common reason for ineligibility was initiation of therapy prior to consultation (61%), often due to clinical urgency. Other frequent reasons included absence of measurable disease (9%) and inadequate performance status (7%) (Figure 2).

### Diagnosis-To-Treatment Interval

To further contextualize trial ineligibility and access patterns, we examined the DTI among subgroups. Trial-ineligible patients had a shorter median DTI of 27 days (IQR 13–48), compared to 36 days (IQR 27–52) among trial-eligible patients who did not enroll and 33 days (IQR 28–43) among those who enrolled. In pairwise comparisons, DTI was significantly shorter for trial-ineligible patients than for trial-eligible patients overall (*P* = .012), consistent with our finding that the most common reason for trial-ineligibility was early initiation of therapy due to clinical urgency. There was no statistically significant difference in DTI between enrolled and non-enrolled trial-eligible patients (*P* = .30).

### Geographic Distribution of Trial Eligibility and Enrollment

County-level mapping of trial eligibility and enrollment revealed that trial-eligible patients were geographically dispersed across the state (Figure 3). However, enrolled patients were predominantly concentrated in counties located near the academic center, while several more distant counties had eligible patients but no enrollments. Trial-ineligible patients were more broadly distributed than eligible patients. Notably, counties farther from the trial site also tended to have higher ADI scores. Together, these patterns illustrate a narrowing geographic funnel from referred patients to those ultimately enrolled, reflecting overlapping geographic and socioeconomic barriers to trial participation.

## Discussion

Our findings demonstrate that geographic distance and, to a lesser extent, neighborhood SES, significantly impact clinical trial enrollment in first-line LBCL IITs at our center. Patients who enrolled in a trial lived significantly closer to the center and had lower ADI scores than those who were eligible but did not enroll. These results underscore the role of both structural and social determinants of health in shaping access to trials, even in IITs designed with broader eligibility and streamlined workflows.

Among trial-ineligible patients, the most common reason for ineligibility (61%) was initiation of therapy prior to consultation, often due to urgent clinical needs prompting treatment in the community. Trial-ineligible patients also resided farther from FHCC and in areas with higher socioeconomic deprivation, notably mirroring the geographic and socioeconomic profiles of patients who were trial-eligible but did not enroll. Although causality cannot be established, this pattern suggests that delays in referral or anticipated logistical barriers, particularly when urgent treatment is warranted, may preclude timely evaluation for trial eligibility. This was reflected in the significantly shorter DTI among trial-ineligible patients. These findings emphasize the need to streamline referral and diagnostic workflows to enable earlier triage and trial consideration. Notably, among trial-eligible patients, enrollment was not associated with treatment delays, with DTI similar between those enrolled and those treated off-trial.

To further explore enrollment predictors, we employed both stepwise logistic regression and elastic net regression. Stepwise analysis identified geographic distance as the only independent predictor. In contrast, elastic net regression, which is more robust in small sample sizes and accounts for collinearity between predictors, identified both distance and ADI as frequently selected variables. This distinction suggests that while ADI may not be an independent predictor once geographic distance is accounted for, it likely serves as a proxy for other unmeasured barriers such as transportation access, digital connectivity, and care navigation, factors that may compound the effects of geographic distance on trial access.

Although not statistically significant, other demographic imbalances, such as race and insurance, may still reflect underlying disparities. For example, all enrolled patients were non-Hispanic, as were 98% of non-enrolled. While this small absolute difference does not represent a meaningful disparity in our modest sample, the trend is directionally consistent with prior work demonstrating underrepresentation of racial and ethnic minorities in lymphoma trials. In a population-based study of over 91,000 patients across NCI-sponsored trials, Casie et al.^13^ found that Black and Hispanic individuals were significantly underrepresented in lymphoma trials relative to their cancer incidence. Although our study is under-powered to detect such differences with confidence, the observed trends warrant further evaluation in larger, multi-institutional cohorts.

Insurance coverage patterns in our cohort also suggest potential underlying referral disparities. Although our institution accepts Medicaid, 92% of enrolled patients in our cohort had private insurance or Medicare, both of which offer relatively comprehensive coverage. In Washington State, Medicaid and uninsured patients make up approximately 26% of the population, yet these groups were significantly underrepresented in our study.^14^ This likely reflects upstream structural barriers—such as limited access to referring specialists, transportation burdens, and socioeconomic constraints—that reduce the likelihood of reaching a tertiary cancer center. These findings highlight that disparities in trial enrollment may arise well before formal eligibility screening and underscore the need for proactive community outreach, navigation support, and decentralized models that reduce logistical barriers.

Our findings complement prior work demonstrating that geographic and socioeconomic disparities affect multiple points along the cancer care continuum.^8, 15–17^ For example, a population-based study in Scotland found that patients in remote or socioeconomically deprived areas had shorter diagnostic intervals but experienced higher one-year mortality, suggesting that post-diagnosis barriers, such as limited access to specialized oncology care, may contribute to worse outcomes.^15^ These findings are particularly relevant to clinical trials, which are often embedded within academic centers and co-located with subspecialty expertise. Limited access to these centers may restrict opportunities to participate in therapeutic trials. Decentralized trial delivery models can help extend access to therapeutic trials beyond traditional academic hubs. Supporting this, Lim et al. evaluated patients already enrolled in therapeutic trials and found no differences in survival or toxicity by distance, suggesting that once enrolled, patients may receive comparable care regardless of geographic location.^18^ Together with our findings, this supports the notion that disparities may manifest primarily upstream, during referral, evaluation, or trial consideration, rather than during the trial itself.

Our results differ from the findings of Nze and colleagues, who found no association between travel distance, neighborhood SES, and trial participation in a large lymphoma cohort at MD Anderson Cancer Center (MDACC).^7^ The authors attributed this to MDACC’s robust institutional infrastructure, including dedicated research nurse coordinators, patient navigators, financial assistance services, multimedia education platforms, and behavioral science programs focused on reducing disparities in trial access. Nearly half of their patients traveled from out of state or internationally, representing a self-selecting population with greater resources and motivation for trial participation. These institutional factors likely help mitigate common enrollment barriers and may explain their high trial participation rate of 30.5%, narrowing disparities by distance or SES.

In comparison, our center serves a more regionally concentrated population, where barriers related to distance and socioeconomic deprivation remain highly relevant. These findings reinforce that trial participation is shaped not only by patient-level characteristics but also by institutional infrastructure and referral ecosystems in a multi-level fashion (Figure 4, adapted from Unger and colleagues^19)^. To address these barriers, we propose targeted interventions such as telemedicine-based pre-screening, direct assistance with transportation and lodging, and improved visibility of clinical trial options. Decentralized and hybrid models of trial delivery, such as those modelled through the Leukemia & Lymphoma Society’s IMPACT (Influential Medicine Providing Access to Clinical Trials) initiative, offer promising approaches to expand trial access through academic-community partnerships and reduce travel burden for underserved populations.^20,21^

This study has several limitations. It is a retrospective, single-center analysis at a large academic center, within a relatively homogenous population that was primarily White, non-Hispanic, privately insured or covered by Medicare, and socioeconomically well-resourced. Enrolled patients resided in neighborhoods within the top decile of SES in the United States (median ADI 8), while non-enrolled patients lived in the top quartile (median ADI 21). While these features limit generalizability, they also highlight that structural barriers to trial enrollment can persist even among relatively advantaged populations. Such barriers may be even more pronounced in more diverse or resource-constrained populations, underscoring the need to validate these findings in larger, multi-institutional cohorts. In addition, the modest sample size may have limited the power to detect subtler associations, and survival or disease-related outcomes were not evaluated. Although ADI provides useful context, it does not capture individual-level socioeconomic factors such as personal income, education, employment status, and social support; thus, our inferences are based on neighborhood-level proxies. Prior work has shown that individual-level SES is an independent predictor of outcomes, even when accounting for neighborhood-level socioeconomic deprivation, highlighting the importance of more granular, patient-level data in future studies.^22,23^ Lastly, given the retrospective nature of this study, we were unable to systematically capture patient or provider-level decision-making regarding trial participation. Future research should explore these factors using mixed-methods approaches, in hopes of designing effective, scalable strategies that advance equitable clinical trial access.

## Conclusion

Geographic distance and socioeconomic deprivation remain key barriers to frontline LBCL trial enrollment, even in the context of IITs designed to promote broader access. The most common reason for ineligibility in our study was early treatment initiation prior to referral, disproportionately affecting patients from disadvantaged and remote areas. Addressing these challenges will require coordinated efforts to enhance early triage, streamline referral systems, and adopt decentralized trial models that meet patients where they are. Future IITs should be designed to reduce logistical burdens by incorporating telemedicine-based triage and consent, allowing local lab and imaging use, partnering with community providers, and supporting transportation needs. Embedding these design elements from the outset can help expand access, promote more equitable participation, and ensure that trials better reflect the populations they aim to serve.

## Figures and Tables

**Figure 1:**
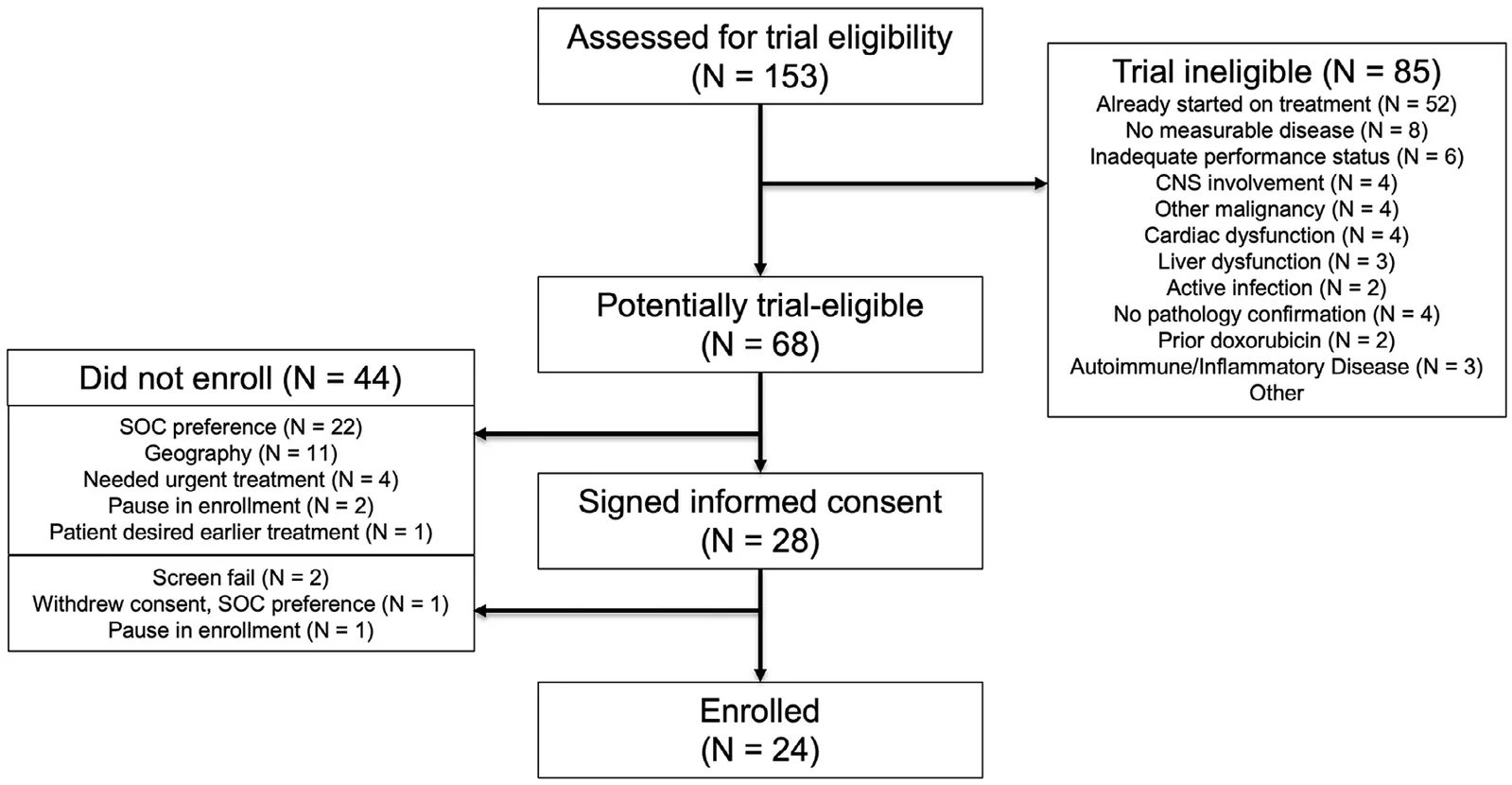
Flowchart of clinical trial screening and enrollment among newly diagnosed patients with large B-cell lymphoma (LBCL). Abbreviations: CNS = central nervous system; SOC = standard-of-care.

**Figure 2:**
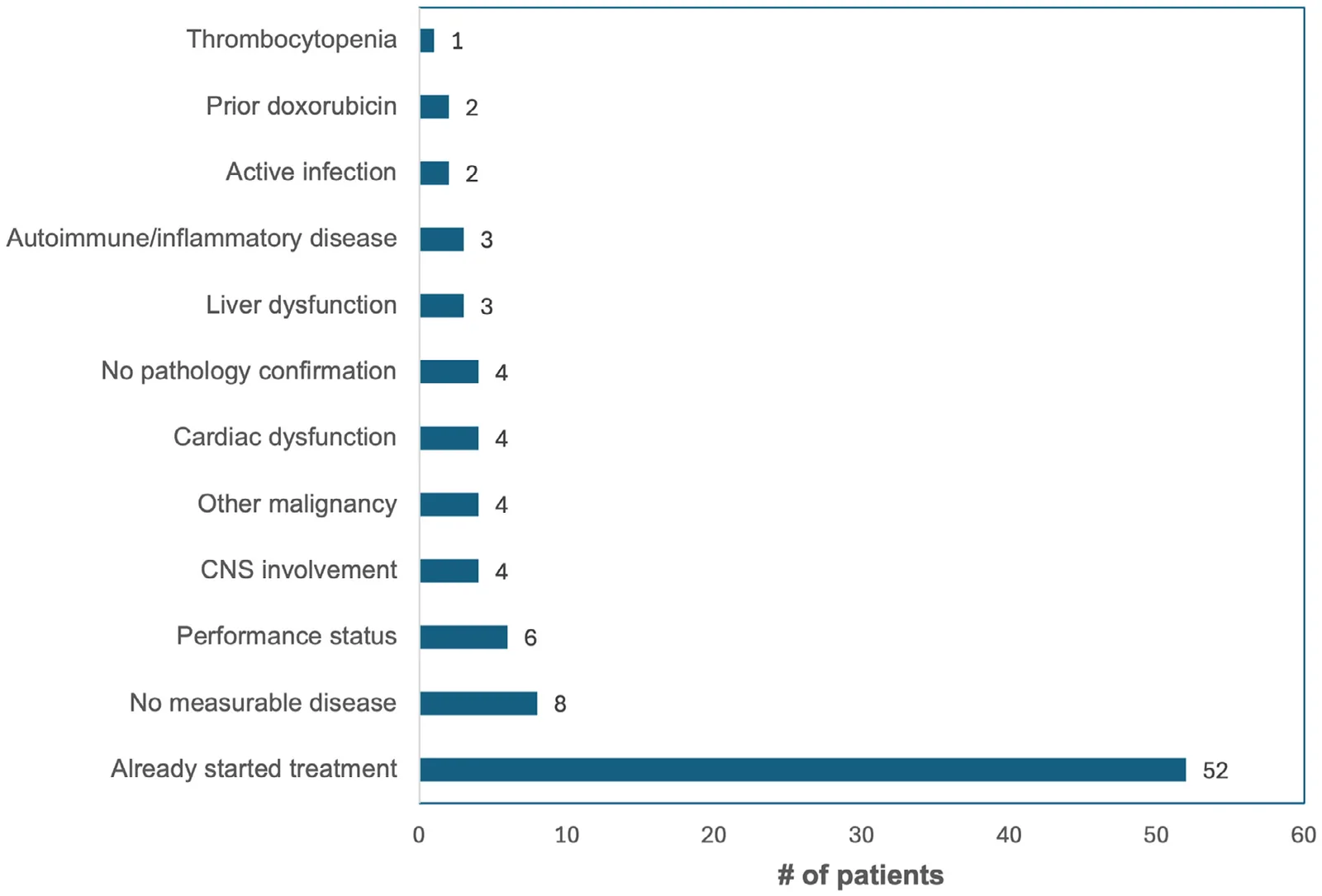
Reasons for ineligibility in trial-ineligible patients (N = 85). Abbreviation: CNS = central nervous system.

**Figure 3:**
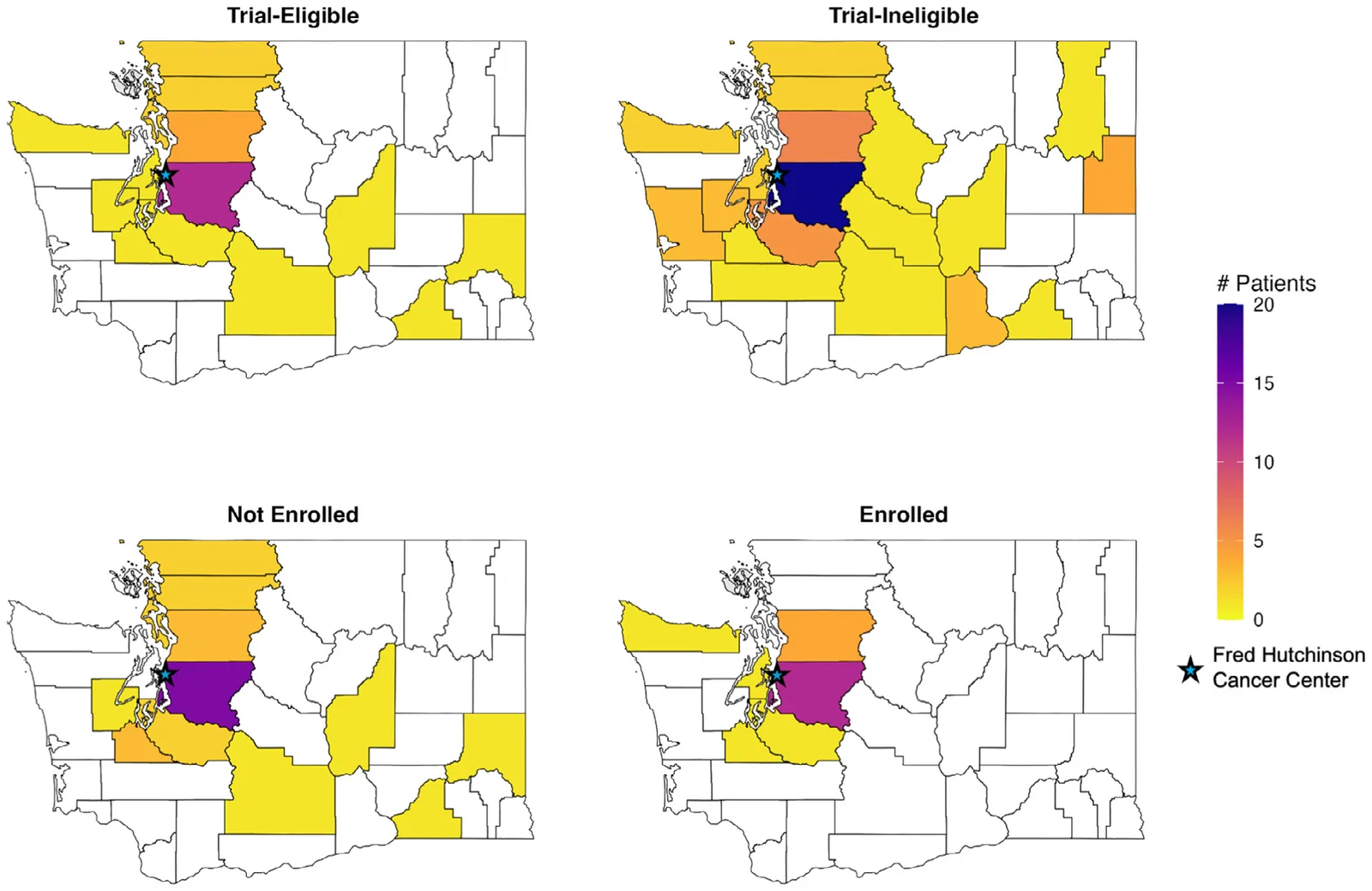
Geographic distribution of patients by trial eligibility and enrollment status. County-level maps show the distribution of patients with newly diagnosed large B-cell lymphoma referred to FHCC, stratified by trial eligibility (top row: eligible vs ineligible) and enrollment status among eligible patients (bottom row: not enrolled vs enrolled). Counties with populations < 20,000 were masked to preserve confidentiality.

**Figure 4:**
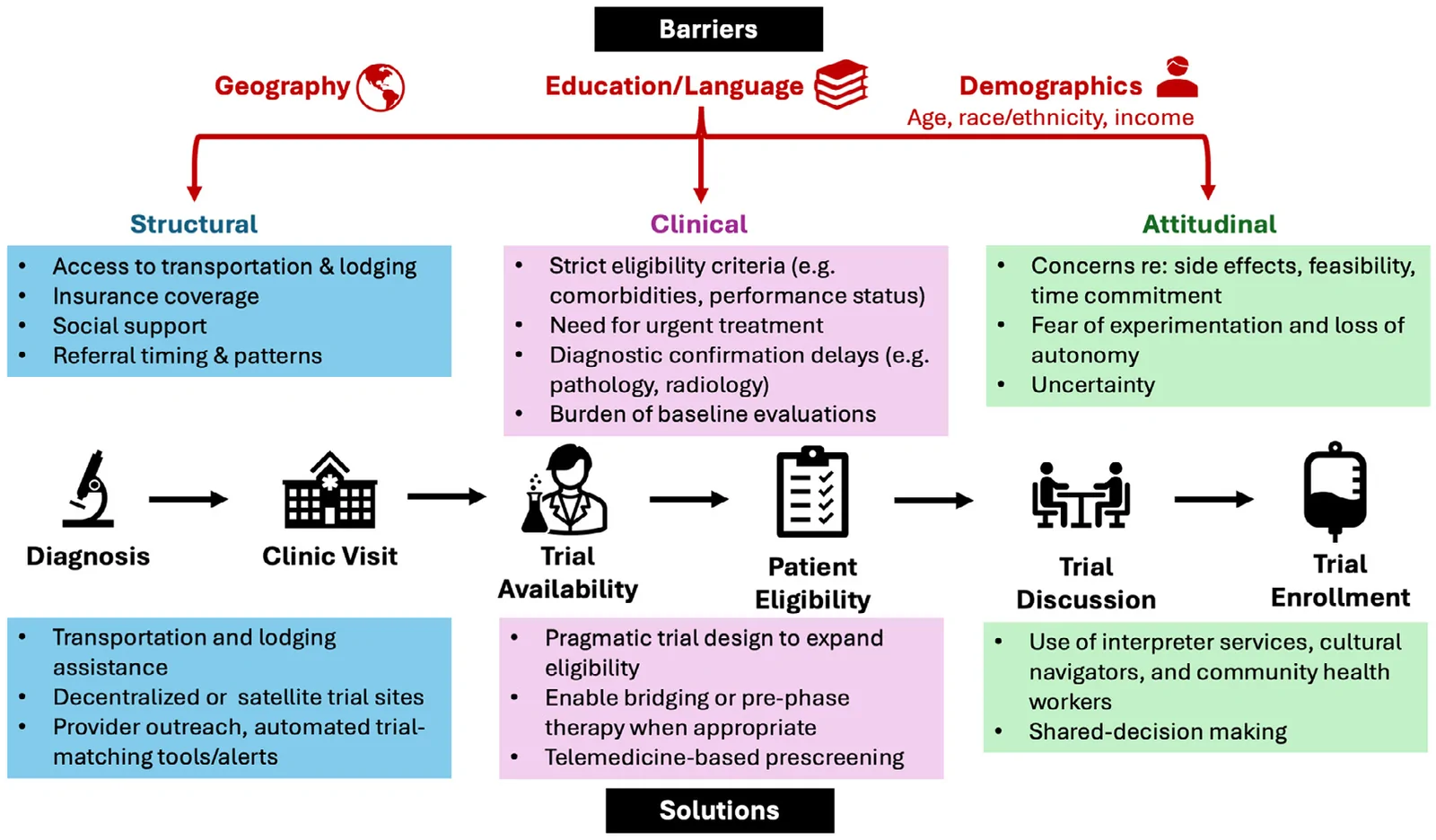
Multilevel barriers to clinical trial enrollment in large B-cell lymphoma. Sociodemographic factors such as geography, education/language, and demographics (eg, age, race/ethnicity, income) influence structural, clinical, and attitudinal barriers throughout the clinical trial enrollment process (framework adapted from Unger et al. [Am Soc Clin Oncol Educ Book 2016]). Proposed solutions target key steps along the patient pathway from diagnosis to trial enrollment.

**Table 1:** Baseline Characteristics of the Study Population (Newly Diagnosed LBCL Patients Referred to FHCC)

Characteristics	Patients (N = 153)
Age, Median (IQR)	67 (53, 73)
Race, n (%)	
White	133 (87%)
Asian	11 (7%)
Black or African American	5 (3%)
Native Hawaiian or Pacific Islander	2 (1%)
N/A	2 (1%)
Ethnicity, n (%)	
Non-Hispanic or Latino/a	149 (97%)
Hispanic or Latino/a	1 (1%)
N/A	3 (2%)
Male sex (%)	94 (61%)
Distance from FHCC (miles), Median (IQR)	50 (16, 130)
ADI, Median (IQR)	21 (8, 31)
Need for interpreter	6 (4%)
Primary insurance	
Medicare	76 (50%)
Private	65 (42%)
Medicaid	10 (6%)
Other government	2 (1%)

Abbreviations: ADI = area deprivation index; FHCC = Fred Hutchinson cancer center; IQR = interquartile range; LBCL = large B cell lymphoma; N/A = not available.

**Table 2:** Baseline Characteristics of Trial-Eligible Patients Who Enrolled Versus Did Not Enroll in a Frontline LBCL Investigator-Initiated Trial at FHCC

Characteristic	Enrolled (N = 24)	Not-Enrolled (N = 44)	*P*-Value
Age, Median (IQR)	58 (39, 69)	66 (52, 72)	.13
Race, n (%)			.78
Asian	1 (4%)	4 (9%)	
Black	1 (4%)	2 (4%)	
Native Hawaiian or Pacific I slander	0	1 (2%)	
White	22 (92%)	36 (82%)	
N/A	0	1 (2%)	
Ethnicity, n (%)			1.0
Non-Hispanic or Latino/a	24 (100%)	43 (98%)	
N/A	0	1 (2%)	
Sex, M (%)	15 (63%)	28 (64%)	1.0
Distance from FHCC (miles), Median (IQR)	15 (7, 18)	50 (8, 111)	.00016
ADI, Median (IQR)	8 (4, 14)	21 (8, 28)	.0045
Need for interpreter, Yes (%)	0	1 (2%)	1.0
Insurance			.52
Medicaid	1 (4%)	3 (7%)	
Medicare	9 (38%)	22 (50%)	
Private	14 (58%)	18 (41%)	
Other government	0	1 (2%)	

*P*-values were calculated using the Wilcoxon rank-sum test for continuous variables and the chi-square test for categorical variables.

Abbreviations: ADI = area deprivation index; FHCC = Fred Hutchinson Cancer Center; IQR = interquartile range; LBCL = large B cell lymphoma; N/A = not available. Bolded values represent statistically significant *p*-values of < 0.05.

**Table 3:** Univariate and Multivariable Logistic Regression: Evaluating Predictors of Trial Enrollment

	Univariate Model		Multivariate Model	
Variable	OR (95% CI)	*P*-value	OR (95% CI)	*P*-value
Distance from FHCC (miles)	0.98 (0.962–0.998)	**.0269**	0.98 (0.962–0.998)	**.0269**
ADI	0.94 (0.899–0.991)	**.0212**		
Sex (Ref = Male)	1.05 (0.375–2.940)	.9260		
Age	0.98 (0.949–1.006)	.121		
Insurance (Ref = Medicaid)				
Medicare	1.23 (0.112–13.425)	.867		
Private	2.33 (0.218–24.923)	.992		
Other government	5.21 × 10^−7^ (<0.001–∞)	.483		
Race (Ref = White)				
Asian	0.409 (0.043–3.9)	.4371		
Black of AA	0.818 (0.07–9.56)	.8729		
Native Hawaiian or PI	2.842 (< 0.001–∞)	.9917		
Need for Interpreter (Ref = Yes)	3.11 × 10^−7^ (< 0.001–∞)	.9918		

Odds ratios (ORs), 95% confidence intervals (CIs), and *P*-values are shown for each variable. Variables with *P*-values < .1 in univariate analysis were included in the multivariable model; *P*-values < .05 were considered statistically significant.

Abbreviations: AA = African American; ADI = area deprivation index; FHCC = Fred Hutchinson Cancer Center; PI = Pacific Islander; Ref = reference.

## Clinical Practice Points

- In a cohort of newly diagnosed LBCL patients referred to a tertiary cancer center, greater geographic distance and higher neighborhood deprivation were strongly associated with lower enrollment in frontline investigator-initiated trials (IITs).
- Early treatment initiation before referral was the most common reason for trial ineligibility, particularly among patients from remote or socioeconomically disadvantaged areas.
- These findings highlight persistent access barriers, even within streamlined IITs at academic centers.
- Expedited referral pathways, telemedicine-based pre-screening, decentralized trial models, and support for transportation and lodging are urgently needed to close access gaps and ensure trials reach patients across geographic and socioeconomic lines.
